# Userscripts for the Life Sciences

**DOI:** 10.1186/1471-2105-8-487

**Published:** 2007-12-21

**Authors:** Egon L Willighagen, Noel M O'Boyle, Harini Gopalakrishnan, Dazhi Jiao, Rajarshi Guha, Christoph Steinbeck, David J Wild

**Affiliations:** 1 Cologne University Bioinformatics Center, Cologne University, Cologne, Germany; 2 Cambridge Crystallographic Data Centre, Cambridge, UK; 3 School of Informatics, Indiana University, Bloomington, USA; 4 Wilhelm-Schickard-Institut, Center for Bioinformatics, University of Tübingen, Tübingen, Germany

## Abstract

**Background:**

The web has seen an explosion of chemistry and biology related resources in the last 15 years: thousands of scientific journals, databases, wikis, blogs and resources are available with a wide variety of types of information. There is a huge need to aggregate and organise this information. However, the sheer number of resources makes it unrealistic to link them all in a centralised manner. Instead, search engines to find information in those resources flourish, and formal languages like Resource Description Framework and Web Ontology Language are increasingly used to allow linking of resources. A recent development is the use of userscripts to change the appearance of web pages, by on-the-fly modification of the web content. This opens possibilities to aggregate information and computational results from different web resources into the web page of one of those resources.

**Results:**

Several userscripts are presented that enrich biology and chemistry related web resources by incorporating or linking to other computational or data sources on the web. The scripts make use of Greasemonkey-like plugins for web browsers and are written in JavaScript. Information from third-party resources are extracted using open Application Programming Interfaces, while common Universal Resource Locator schemes are used to make deep links to related information in that external resource. The userscripts presented here use a variety of techniques and resources, and show the potential of such scripts.

**Conclusion:**

This paper discusses a number of userscripts that aggregate information from two or more web resources. Examples are shown that enrich web pages with information from other resources, and show how information from web pages can be used to link to, search, and process information in other resources. Due to the nature of userscripts, scientists are able to select those scripts they find useful on a daily basis, as the scripts run directly in their own web browser rather than on the web server. This flexibility allows the scientists to tune the features of web resources to optimise their productivity.

## Background

The web has seen an explosion of chemistry and biology related resources in the last 15 years: thousands of scientific journals, databases, wikis, blogs, and regular HTML pages are available containing information relevant to chemists and biologists [1-4]. While each of those resources is valuable in itself, integrating information from these resources increases the value even more: for example, PubChem provides a wealth of data but could be complemented with 3D models to create an even richer information source.

The original goal of the world wide web was to hyperlink individual web pages allowing humans to explore a web of knowledge. For individual web pages these links can be created manually, as is still done in blogs, wikis, and static HTML pages; for large databases this is, however, not feasible. Userscripts are small programs that can alter the HTML content rendered by web browsers. For example, a userscript may add book prices from competitors to the Amazon.com website, or may remove unwanted advertisements from a site. Using the same approach, userscripts can also solve the problem of interlinking web resources, by adding to web pages of one resource dynamically generated hyperlinks into another. By selecting a specific set of userscripts, the user can tune a website to provide all kinds of facilities not anticipated by the original author of the site. For example, userscripts have been used in bioinformatics to enhance the iHOP web page [5]: the script extracts user assigned tags from a third party resource, and shows them as a tag cloud on iHOP pages for particular genes.

Automatic hyperlinking is only possible though the use of unique identifiers such as the PDB ID, the CAS registration number and, more recently, the IUPAC International Chemical Identifier (InChI). While identifiers are easily used to connect databases, such as done in the SRS system [6] or in meta database software like BioWarehouse [7], the sheer number of web resources makes it impossible integrate all resources. Consequently, (bio)chemical search engines, such as ChemSpider [8] and tools to harvest information from web resources, such as ChemXtreme [9] and BioSpider [10,11], as well as systems that standardize algorithmic access to resources and services, such as BioMOBY [12], have emerged.

Another reason why identifiers do not always allow linking resources is that many of them are database specific, such as the PDB ID and the Digital Object Identifier (DOI), and sometimes even restricted in being used, as with the CAS registration number. Open standard identifiers address this problem. Such identifiers can be derived from ontologies, dictionaries, encyclopedia, or computed by an algorithm. The Gene Ontology terms are often used as identifiers [13], indicating that a specific database entry is related to the cited term in the ontology, and therefore related to entries from other databases annotated with that term.

Identifiers that can be calculated algorithmically are even better, because they do not need to be looked up in a list of identifiers. Instead, anyone can calculate them from the object itself. For example, for molecular structures the InChI [14] is the ideal replacement for database specific identifiers such as the CAS registration number, the PubChem compound identifier and the ChEBI identifier. These all require a look up or conversion table to convert one identifier into another. Using the InChI, one can look up information in all databases without having to know the database specific identifier.

In addition to the unique identifier, one additional functionality is needed to create a link to a particular database: the database must provide either an API (Application Programming Interface) which can be queried using the identifier or else provide a uniform scheme for deep linking to a web page containing information about the entry behind the identifier. For example, looking up structures in PubChem is done with a scheme in which the InChI is embedded verbatim. To look up the structure of methane (InChI=1/CH4/h1H4), the URL  is used.

The plethora of resources is overwhelming, and both users and database developers may have preferred subsets, e.g. more trusted, resources. It is therefore worthwhile to have a system that allows users to choose which resources they want to have linked with which other resources. Userscripts provide the necessary technology to allow this within web browsers. Here we describe several userscripts we have developed to create links between web resources of interest to researchers in the life sciences.

## Implementation

We use the following techniques to link various web resources in this paper: userscripts, unique identifiers, microformats, and web resource interfaces. The following sections describe how these are used in this work.

### Userscripts

A userscript is a small program written in JavaScript that is automatically run within a web browser (often by a plugin or add-on) when the user accesses pages that match a particular URL. Userscripts allow the user to modify the HTML content of a web page on-the-fly, by adding or removing elements or by moving them around. For example, userscripts exist that remove pop-up advertisements from web pages, and that alter the Amazon.com web page to provide book prices from alternative suppliers. A repository of userscripts exists at userscripts.org/citeuserscriptsdotorg. Chemists and biologists can find relevant userscripts by searching with the terms "chemistry" or "biology".

Of the popular web browsers, only Opera provides built-in support for userscripts (referred to as 'User JavaScript'). To enable userscript support in other browsers, a third-party extension needs to be installed: Greasemonkey [15] for Firefox, Creammonkey [16] for Safari, IE7pro [17] or Turnabout [18] for Internet Explorer. The userscripts presented in the Results section are targeted at Greasemonkey, although it should be possible to run them in any browser with only minor changes.

The web browser user has full control over which userscripts she wants to have installed, allowing her to customise web pages exactly the way she wishes. Once installed, it is possible to individually enable or disable installed scripts. For example, for Greasemonkey see the "Manage User Scripts" option in the Tools menu under "Greasemonkey", or to disable the extension completely, click on the Greasemonkey icon in the status bar. Further control is provided by specifying to which web pages the script applies. Userscripts define default rules (e.g. http://www.biomedcentral.com/), but the user is normally able to override these.

The userscript has two main methods to find the HTML content to which to add or remove elements. The most accurate one is to analyse the document object model (DOM). This approach is used by the Sechemtic userscript to find uses of chemical microformats (see example below under Microformats). The other method is to use regular expressions to find certain strings in the text of the web page. This works particularly well for identifiers with a unique and well described syntax. For example, a regular expression for InChIs will have fewer false positives than one for PDB identifiers.

As with any program that you run on your computer, it is important to consider security when installing userscripts. Although the security model used by Greasemonkey prevents attacks by malicious websites, it is unable to detect or prevent the user himself installing a malicious userscript. Such scripts do exist; recently, malicious userscripts were uploaded to Userscripts.org that attempted to steal information from users' cookies. In that case, once the problem was discovered the malicious userscripts were easily detected and removed by the administrator. We recommend that unless you are familiar with JavaScript and carefully inspect the source code, you should only install userscripts from a trusted source.

### Unique identifiers

Recognition of biological and chemistry relevant information on web pages is simplified by using identifiers [19]. Such identifiers may or may not be marked up with semantic markup such as microformats (see below). Identifiers are widely used to make connections between databases, and often identify a specific entry in a database. Some examples of this are the PDB identifier, Digital Object Identifiers, PubChem compound identifier, and the CAS registry number for, respectively the PDB, DOI, PubChem, and the Chemistry Abstract Service databases. In this study we use DOIs, InChIs, and PDB identifiers as our unique identifiers (Table 1).

**Table 1:** Userscripts for the life sciences. A summary of the resources and identifiers used by userscripts for the life sciences. The Identification method indicates how the userscript recognises relevant information on a web page. The Identifiers column describes the unique identifier searched for. The Resources column indicates the web resource to which a link is created, or from which data is extracted.

	Technologies and Resources used		
Name	Identification method	Identifiers	Resources

Jmol4PubChem	HTML tags on PubChem	PubChem ID	Pub3D [36]
OSCAR3 on HTML	natural language processing	chemical structure name	-
PDB-Jmol	regular expression	PDB ID	First Glance in Jmol [41]
Sechemtic	microformats	InChI, SMILES, CAS number	PubChem [32] eMolecules [43] Google [54]
Add quotes to DOIs	regular expression	DOI	Postgenomic [3] Chemical blogspace [4]
Add quotes to molecules	microformats	InChI	Chemical blogspace [4]
Add to Connotea	regular expression	DOI	Connotea [30]

### Microformats

Microformats [20] are a lightweight specification that extends HTML to add semantic markup to web pages. For example, hCard is a microformat that allows semantic mark up of address information [21], and hCalendar is a microformat specification for the representation of calendar information about events [22].

A microformat specification has also been suggested for chemistry that would make it much easier to recognise compound names, InChIs, SMILES and CAS registry numbers. Userscripts, or indeed any other programs, would then no longer need to depend on regular expressions to find names and identifiers, but could use this markup to accurately extract the identifier.

For example, a web page implementing the InChI microformat would wrap any InChIs in a HTML <span> element with a @class attribute as follows: <span class="inchi">InChI=1/</span>. This information can easily be extracted using the document.evaluate method which takes an XPath [23] expression (//span[@class="inchi"] in this case):

allInChIs = document.evaluate(

   '//span[@class="inchi"]', document, null,

   XpathResult.UNORDERED_NODE_SNAPSHOT_TYPE,

   null

);

This code returns all HTML nodes that mark up InChI strings using the InChI microformat. By iterating over these nodes, the userscript can insert new HTML elements, such as links to external resources as shown here in code taken from the Sechemtic userscript:

for (var i=0; i<allInChIs.snapshotLength; i++){

      spanElement = allInChIs.snapshotItem(i);

      inchi = spanElement.innerHTML;

      // create a link to PubChem

      newElement = document.createElement('a');

      newElement.href = "http://www.ncbi.nlm." +

         "nih.gov/entrez/query.fcgi?CMD=search" +

         "&DB=pccompound&term=%22" + inchi +

         "%22[InChI]";

      newElement.innerHTML =

         "<sup>PubChem</sup>";

      spanElement.parentNode.insertBefore(

         newElement, spanElement.nextSibling

      );

}

### Web resource interfaces

Web databases are the primary source of information used by the discussed userscripts. While it is easy to have scripts create links to external web resources, it is also possible for them to retrieve information from those resources and include it in the HTML content of the web page the user is browsing. The latter is, for example, performed by the userscript that adds comments from Postgenomic.com and Chemical blogspace to journal web pages.

The general approach userscripts use to retrieve information from external web resources uses HTTP just like any web browser itself. To simplify the process, userscripts tend to use a combination of *XMLHttpRequest*, possibly via the Greasemonkey *GM_xmlhttpRequest *wrapper method, and the JavaScript Object Notation (JSON) format [24] for data representation. The XMLHttpRequest method retrieves the information using a URL that normally points to a data interface, or API. The Postgenomic.com software has such an API that returns the blog posts that discuss a particular article, as identified by its DOI. Chemical blogspace uses the same API, and adds another one to return blog posts that discuss a particular molecule, as identified by its InChI. Both database APIs can return the information as JSON objects, which is how they are used in the discussed userscripts.

Since our userscripts rely on a particular API or specially-constructed URL to access an external resource, they will fail if the external resource changes its API or the URL it provides to access it. This will not affect the browsing experience of the user, but the additional functionality provided by the userscript will no longer be available. To deal with this, each of the userscripts described in this article checks once a day for a new version and prompts the user to install it if one is available. This means that when a userscript is updated to deal with a new API or URL, every user will quickly have access to the latest version.

## Results

This paper introduces userscripts that have been written in our research groups as exemplars of how web resources can be integrated and to outline how they can be used in research. Our userscripts can be classified into two broads areas: those that link chemical and biological data to websites, and those that affect how we interact with the scientific literature.

In the following sections, we describe in detail how functionality is added to the web page being browsed. Table 1 summarises the resources linked to, or accessed, by each script, as well as the unique identifier used.

### Interacting with the scientific literature

#### OSCAR3 running on HTML

Published journal articles and other web documents with chemistry content are not normally marked up by the publishers or authors to provide machine readable representations of chemical structures and related information. As a result, there has been active interest in methods which can mark documents up automatically. In particular, OSCAR3 [25,26], developed at the Unilever Centre for Molecular Informatics at the University of Cambridge, and used by the Royal Society of Chemistry in their Project Prospect [27], searches documents for chemical names, spectra, and other chemical information, and automatically marks up the content using XML tags (to the extent of where possible generating machine readable SMILES and InChI structures for chemicals referenced in the document).

**Figure 1:**
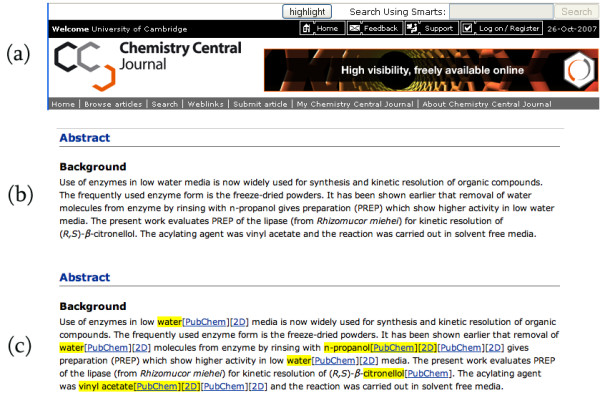
**Highlighting and annotating chemical terms in an online journal article**. Screenshots showing the effect of the ChemGM.user.js userscript on the *Chemistry Central Journal *web page (full URL: [47]) for Majumder et al. [48]. (a) When the userscript is running a toolbar is added to the top of every webpage. Clicking the highlight button in the toolbar causes the contents of the webpage to be analysed for chemical terms. (b) shows the original text of the abstract. (c) After a minute or so, any chemical terms recognised are highlighted in yellow, and are annotated with hypertext links to their entries in PubChem (if available) and a 2D depiction of the image.

We have created a userscript, ChemGM.user.js that will automatically run OSCAR on a web page and provide inline hypertext links to PubChem for chemical structure names that are found in the page (including 2D structure depictions generated by another web service and PubChem searches). The userscript can be run on any web page, but it is particularly applicable to online journal articles and chemistry blogs. An example highlighting the effect of this userscript is shown in Figure 1. Note that though the images use an article from *Chemistry Central Journal*, the script can be applied to any web page, irrespective of its source or content.

#### Add quotes from Chemical blogspace and Postgenomic to DOIs

It can be a challenge to keep up with the primary literature in a field. At the same time, there are a large number of scientific blogs, many of which have reviews of the recent literature or highlight interesting papers. The Postgenomic web site was developed by Euan Adie and later hosted by Nature Publishing Group and currently aggregates information from over 750 scientific blogs [3]. The source code is open and has been used by one of the authors (ELW) to establish a similar site, Chemical blogspace, for over 140 blogs with chemical content [4]. Both of these sites identify references to journal articles in blogs, and make this information available through an API. Compared to the Postgenomic website, the Chemical blogspace site also identifies molecules referenced in blogs either by microformat markup of InChI and SMILES, or by analysing links to Wikipedia [28]. If the latter link points to a wiki page that contains a PubChem compound identifier or an InChI, then the molecular structure is linked to the blog post.

**Figure 2:**
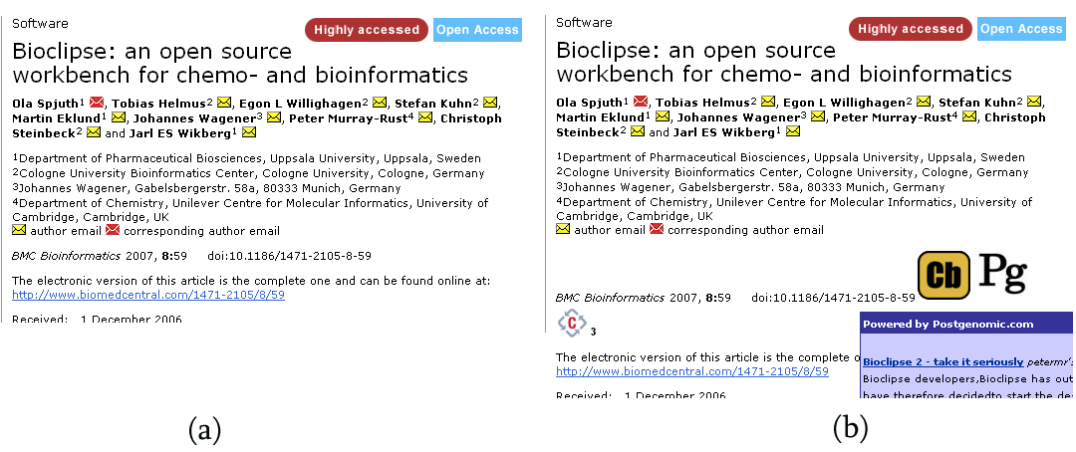
**Adding information to DOIs on journal web pages**. Screenshots from the *BMC Bioinformatics *web page (full URL: [49]) for Spjuth et al. [29] (a) without any userscript enabled, and (b) showing the effect of the two userscripts "Add quotes from Chemical blogspace and Postgenomic to DOIs" and "Add to Connotea". The latter added a Connotea logo (a 'c' surrounded by linking arrows), which links to the Connotea dialog box for adding this paper to your library, and a number indicating how many people have already bookmarked this paper, which links to the existing entry for this paper on Connotea. The "Add quotes" userscript added the Cb logo, which links to the Chemical blogspace page for this paper, and a Pg logo, linking to the Postgenomic page. The popup titled "Powered by Postgenomic.com" (only partially shown) appears when the mouse is placed on the Pg logo, and contains quotes from and links to the citing blog articles.

This userscript uses the aggregated information collected by Postgenomic and Chemical blogspace. It runs whenever the user accesses the website of a journal publisher. It identifies any DOIs on the page, and uses the Chemical blogspace and Postgenomic APIs to find out whether those DOIs have been referenced in a blog post. If so, an icon is added to the web page next to the DOI which, if hovered over with the mouse, causes a popup to appear containing the name of the citing blog post, the blog name, and the first few lines of text of the blog. The full content can be accessed by clicking on the title of the blog post. In this way, content from blog articles widely dispersed in terms of the web is brought directly to where it is likely to be of most interest – the journal web site. Figure 2 shows the effect of this userscript when running on the HTML version of Spjuth et al. [29].

Providing reviews of journal articles is only one of the uses of such a userscript. It is also a general way to create a link between the content of a blog post and a particular paper. In this way, bloggers can use blog posts to enhance the original journal website without any intervention required by the publisher. For example, the author of a paper may write a blog post which provides additional supporting information for a journal article or includes the article preprint for those who do not have a subscription. Alternatively, the author of a paper may write a blog post and include the DOIs of all of the references. This would not only promote his/her own paper (all of the cited papers would show a blog comment pointing to the citing paper), but would result in an eventual network of citations which could be used to measure the impact of a paper.

#### Add to Connotea

Connotea is a social bookmarking site developed by Nature Publishing Group for scientists [30]. It allows a user to bookmark websites using either the DOI or a URL, and to tag those bookmarks. Crucially, it also provides an API for retrieving information.

The "Add to Connotea" userscript has two aspects. Firstly, it makes it easy to add papers to Connotea from journal webpages, by adding a hyperlink in the form of the Connotea logo next to every DOI identified on a journal web page. Clicking on the logo brings the user to the Connotea page for adding new papers. This aspect of the userscript is not entirely novel. A userscript has previously been developed which allows the user to add papers to Connotea from NCBI PubMed [31]. In addition, a small number of publishers (which includes BioMed Central and Nature Publishing Group), provide a facility to add papers to Connotea directly from their website. Our userscript differs in that it will work on the website of *any *journal publisher where the text contains DOIs.

The second aspect of this userscript is more interesting in the context of this paper. The userscript queries the Connotea API to find out how many people have previously added this paper to their Connotea account. It then adds this number next to the Connotea icon. Clicking on the number brings you to the Connotea page for that paper. From here it is possible to access comments on the paper. More useful perhaps, is the ability to find related papers by looking at the other papers a particular Connotea user has tagged with the same tag. Figure 2 shows the effect of this userscript when running on the HTML version of Spjuth *et al.*[29].

This aspect of the userscript has the potential to affect the way we read the literature. The number of times a particular paper has been bookmarked on Connotea can be considered a measure of its importance or its interest. In the past, measures such as the number of citations have served this purpose, but this information is generally not shown on journal web pages as it is not freely available. Another effect of this userscript is to link the paper the user is viewing to related papers through the Connotea website. If a researcher finds that a particular paper has been bookmarked on Connotea and is of interest to him or her, he or she can is likely to find other relevant papers by browsing through the other papers bookmarked by the same Connotea user with the same tag.

On a technical note, this userscript illustrates some techniques necessary for accessing an API that requires a user name and password and that, in addition, only permits one API request every two seconds or so. Note that this userscript requires the user to have a Connotea account (which is freely available at Ref. [30]).

### Linking to chemical and biological data sources

#### Enhancement of PubChem with 3D structures

The PubChem repository is a public collection of over 10 million compounds [32]. The database contains 2D structures as well as a number of precomputed properties (such as number of heavy atoms and topological polar surface area [33]). The web interface to this database allows a wide variety of queries. The results are usually represented in the form of a summary web page containing images of the 2D structures of all the compounds satisfying the query with links to pages for individual compounds which provide a summary of the properties of the compound. In many cases it would be useful to be able to view an image of the 3D structure of a molecule. However, PubChem currently does not contain 3D structures for the compounds stored in the database.

To address this problem, we developed a database of 3D structures of PubChem compounds as part of our web service infrastructure for chemoinformatics [34]. The structures were generated using a two-step process in which the SMILES were converted to a set of rough 3D coordinates using stochastic proximity embedding [35] and subsequently geometry optimised using the MMFF94 force field, using in-house code. A number of compounds were excluded from the final 3D database since the force field did not contain parameters for certain atom types. However the 3D database, known as Pub3D [36], contains approximately 99% of the compounds in PubChem. Pub3D is wrapped by a set of web services which encapsulate common queries including finding a structure by compound ID (CID) or finding structures matching a SMARTS pattern.

**Figure 3:**
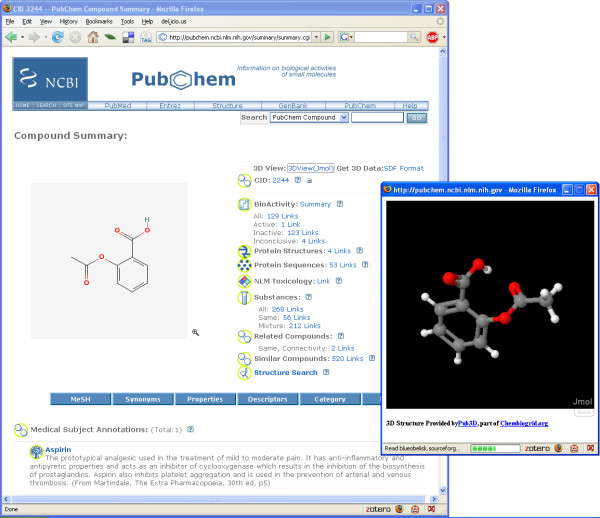
**Adding 3D models to PubChem**. Screenshot of the PubChem web page for aspirin (full URL: [50]) with the "3DStructureView" userscript enabled. The userscript added the first line of text in the compound summary information. Clicking on the "3DView(Jmol)" link causes a window to popup showing a 3D model of the structure. Clicking on the "SDF Format" link allows the user to download the calculated 3D structure of the molecule in SDF file format.

Using this web service interface we created a userscript called 3DStructureView.user.js that allows 3D structures from our database to be shown when users visit the PubChem website (see Figure 3). The script is designed to work only on the summary and detail pages that a user views after a PubChem search. It parses the page and identifies the compound ID which is then used to construct a call to the Pub3D database. The return value is a string containing the 3D structure of the compound, in SD format, which is used to construct an appropriate URL. The result of this process is that the user can now click on a link titled *3DView(Jmol)*, which will cause a Jmol applet [37-39] window to appear showing the 3D structure of the compound in question.

As an example, after installing the script, one can navigate to the PubChem website [32] and search for entries related to aspirin. This should return slightly more than thirty hits. If one then clicks on the compound ID for the first hit, one is taken to a summary page which provides various details regarding the molecular structure and biological activity of aspirin. In addition to the data provided by PubChem, the userscript has enhanced the page to add two links: *3DView(Jmol) *and *SDF Format*. The former link will bring up an instance of the Jmol applet showing a 3D structure of aspirin, while the second link allows one to download the 3D structure in the SD file format (see Figure 3).

#### PDB-Jmol Greasemonkey Script

**Figure 4:**
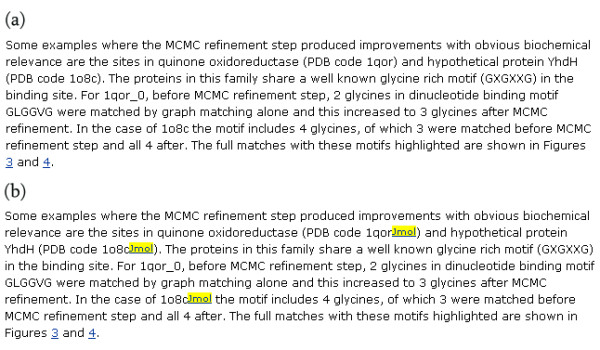
**The effect of the PDB-Jmol userscript**. Screenshots from the *BMC Bioinformatics *web page (full URL: [51]) for Mardia et al. [42] showing a paragraph containing PDB identifiers (a) without the PDB-Jmol userscript installed, and (b) with the PDB-Jmol userscript installed. The "Jmol" text in yellow is a hyperlink to the FirstGlance in Jmol page [41] for a particular protein structure.

The Protein Data Bank [40] is a repository of experimentally-determined 3D coordinates of proteins. Each entry has a PDB ID, which is a unique four letter identification code consisting of a number followed by three characters which can be either letters or numbers; for example, 1abe, 114L and 6NN9. The PDB-Jmol Greasemonkey userscript identifies all PDB IDs on web pages and adds hyperlinks to the FirstGlance in Jmol web page [41] for that protein. This website uses the Open Source molecular viewer Jmol to show the protein as a 3D model which can be manipulated by the user. In this way, the user can instantly view the 3D structure of any PDB ID mentioned on a website, and in particular, if the user is reading the HTML version of a journal article on-line, all PDB IDs in the paper will similarly be enhanced. Figure 4 shows an example of the latter case where PDB identifiers in the the online version of Mardia et al. [42] have been identified and links added.

As this userscript runs on all web pages accessed by the user, and since the search term is simply 4 characters long, additional constraints are necessary to prevent excessive false positive identification. The userscript only looks for PDB IDs if it finds one of the following terms in the web page: "protein", "PDB", or "enzyme".

#### Sechemtic

**Figure 5:**
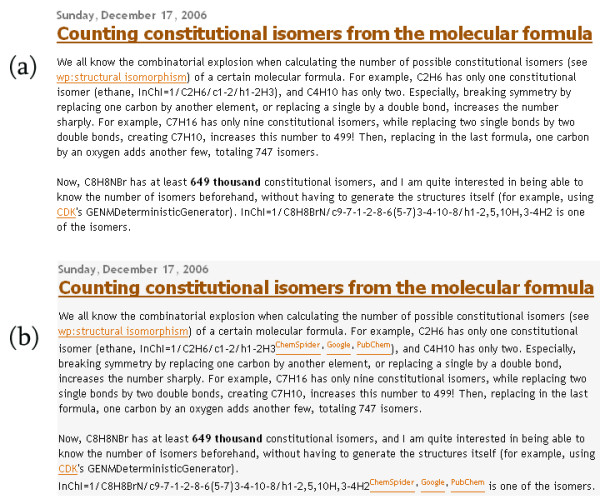
**Annotating chemical terms marked up with microformats**. Screenshots showing a blog post (full URL: [52]) containing chemical terms marked up with chemical microformats, (a) without and (b) with the "Sechemtic" userscript enabled. The added hyperlinks allow the user to look up the structure in Google, ChemSpider and PubChem.

Sechemtic is a small userscript that detects use of microformats (see Implementation) to markup molecular identifiers, as well as regular molecular names. It recognises markup for the IUPAC InChI and SMILES, and creates links for those molecules to web resources like eMolecules [43], PubChem [32] and a link to Google to search for more information (see Figure 5). It should be noted that, in particular with Google searches, the links based on InChIs are more useful as the same molecule may be represented by several different SMILES strings but only a single InChI.

From a technological point of view, these scripts are very simple in nature; the semantic nature of the (chemical) microformats is what makes this simple script possible. The semantic markup in HTML for InChIs that is picked up by the userscript looks like <span class="inchi">InChI=1/CH4/h1H4</span> while the markup for a SMILES string looks like <span class="smiles">CCO</span>.

#### Add quotes from Chemical blogspace to molecules

**Figure 6:**
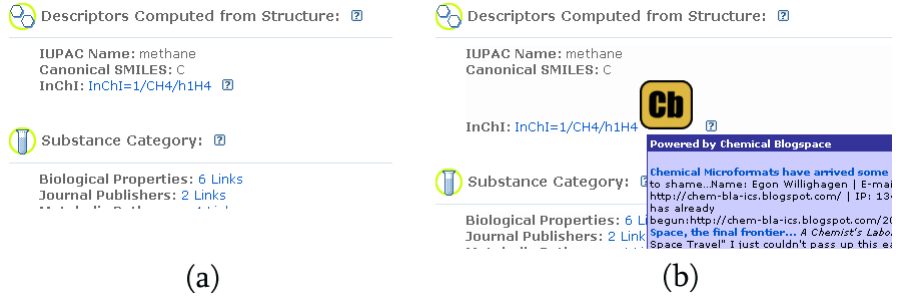
**Adding comments from the blogosphere to molecules**. Screenshots from the PubChem web page for methane (full URL: [53]), (a) without and (b) with the "Add quotes from Chemical blogspace to molecules" userscript enabled. The InChI *InChI=1/CH4/h1H4 *is identified by the userscript, which then adds the Cb logo. The logo is a link to the Chemical blogspace page for this molecule. The popup titled "Powered by Chemical blogspace" (only partially shown) appears when the mouse is placed on the Cb logo, and contains quotes from and links to blog posts that discuss this molecule.

This userscript, quite similar to the one that adds comments to DOIs, runs on all web pages accessed by the user. Using the same method as the Sechemtic userscript (see above), it identifies any molecules referenced on a page which have been marked up with the appropriate tags. It also supports the (non-marked up) InChI tags on PubChem. It then uses the Chemical blogspace API to find out whether this molecule has been referenced in a blog post. The remainder is as for the previous userscript; an icon is added which contains a popup to the citing blog post. Figure 6 shows the effect of the userscript on the PubChem page for methane (InChI=1/CH4/h1H4). A full list of molecules with comments in Chemical blogspace is available from Ref. [44].

A possible use of this script is to link all discussions of a particular drug in the blogosphere to a static page containing information on the drug. Another use is to link discussions on syntheses of molecules to pages containing references to the molecule.

## Discussion

Here we have focused on the development of userscripts that enhance web pages for biologists and chemists. If all of these userscripts are installed, any web page with a PDB code will now contain a link to view the structure in 3D, journal webpages will show chemical structure markup and blog comments on articles, 3D structures and links to appropriate blog posts will be available from PubChem, and any use of chemical microformats will be picked up adding links to Google, eMolecules, ChemSpider and PubChem.

These examples show that userscripts offer a powerful technology to improve the way we read the scientific literature and access (bio)chemical databases. This is done by dynamically combining web resources, and enriching the information content of the primary resources. Theoretically, such links can be made on the web server itself, and this is commonly done, but it does not give the user the flexibility to choose what features to install. The crucial point about userscripts is that they do not require the involvement of the web site provider. All of the enhancements are done on-the-fly by the user's browser.

The userscripts combine a number of technologies for data retrieval and communication. Information from HTML pages is extracted using identifiers, regular expressions, XPath queries and microformats. It is noted that the syntax of (bio)chemical and other identifiers is generally not distinct enough to detect them with perfect recall and optimal precision. It is easiest to write regular expressions for the DOI and the InChI with a high precision, compared to, for example, the PDB ID which has a syntax which can clash with other web page content.

Microformats offer a solution for such less well-defined identifiers. This technology is used to wrap identifiers with some semantic markup so that the userscript can easily extract the identifiers using XPath queries. However, microformats do not incorporate a mechanism to provide details on what a microformat means. That is, microformats are not backed up by a specified ontology. As a result the chemical 'smiles' microformat, to markup SMILES, may collide with a microformat specification to markup moods.

Once the identifier is extracted by whatever means, the userscripts can either create links to other web resources, or query those resources and embed results into the HTML of the web page on which the userscript is run. While any HTTP-based approach can be used for this, the example userscripts show that combining XMLHttpRequest with JSON [24] is a rather straightforward approach.

## Conclusion

We have shown that userscripts are a simple and useful way of integrating bio- and chemoinformatics web resources. In particular, they permit (a) the augmentation of existing websites with functionality not envisioned or indeed wanted by the original author, (b) the integration of information from different domains, and (c) a connection point between the social web (wikis, blogs etc.) and traditional web tools and sites. We continue to find interesting uses for userscripts, and we hope this manuscript will spur others to do likewise.

## Availability and requirements

• **Project name: **Userscripts for Chemistry and Biology

• **Project home page: **Blue Obelisk [45] website [46]. Download link: 

• **Operating system(s): **Platform independent

• **Programming language: **JavaScript

• **Other requirements: **Firefox with Greasemonkey add-on (or equivalent) for userscript support; Java is required to view the Jmol applet; a Connotea account is required for the Add to Connotea userscript

• **License: **GNU GPL, BSD

• **Any restrictions to use by non-academics: **none

## Authors' contributions

NMOB, ELW, HG and DJ have written userscripts mentioned in this text. RG developed and maintains the 3D structure database and contributed to the development of the Pub3D userscript. DW and CS devised and tested some of the userscripts. All authors have read and approved the final manuscript.
